# Strangling the *White Lotus*: The dense psychodynamic roots of modern media

**DOI:** 10.1177/10398562231195440

**Published:** 2023-08-18

**Authors:** Andrew James Amos

**Affiliations:** 104397 Townsville, QLD

Dear Editor,

Rorschach inkblots elicit the projection of unconscious dynamics, neutralising defences by their provocatively amorphous shapes. Modern TV often does the opposite, functioning as holding environments that contain and anaesthetise audiences’ unconscious anxieties with comforting fantasies. *The Sopranos* humanised psychopaths, reframing criminals from universally dangerous beasts to rational actors preying on the morally weak. *Game of Thrones* leveraged the fear that those in authority are driven entirely by self-regard to sell the fantasy that even bastards and dwarves can triumph over evil if they are true to themselves.

The HBO series *White Lotus* is a black comedy series named for the international chain of exclusive resorts where the action occurs. Its psychodynamic boundaries emerge in the very first scene in which an entitled, wealthy white man returning from an island honeymoon overlooks a body being loaded into a plane, alone. In place of grief, he projects a resigned hostility somewhere between anger and guilt. Within this ambiguous existential frame, the second scene jumps to the beginning of the honeymoon in flashback. The man and his new wife are travelling to an island resort, accompanied by two cynical teens who judge their fellow passengers with a detached superiority that embodies Wilde’s diagnosis of knowing ‘the price of everything, and the value of nothing’.

At the resort, the waiting manager prepares a new staff member with an overtly analytic interpretation:

“You don’t want to be too specific as a presence, as an identity…it’s a Japanese ethos where we are asked to disappear behind our masks, as pleasant, interchangeable helpers… the goal, for the guests, is to create an overall impression of vagueness, that can be very satisfying, where they get everything they want, but they don’t even know what they want…”

Soon after their arrival, when one of the guests complains, the manager goes deeper:

“You have to treat these people like sensitive children…they just need to feel seen…they wanna be the only child…and we are their mean mummies, denying them their [pleasures]”

The tension between this dreamlike environment and the inevitability of death established in the opening scene heightens the various character-driven conflicts. Class conflict is introduced in an early contrast between the wealthy man’s aggressive and his less wealthy wife’s passive responses to a room mixup. This divide is reinforced when the teen judges reject her friendship and her worth as anything other than an appendage to her wealthy husband.

*White Lotus* rides the contrast between the unconstrained lives of the wealthy and the anxieties beneath the pleasant masks that maintain their illusions. The island setting and an early scene with a pregnant staff member suggests the conscious creation of a womblike container within which the creators play with expectations, bypassing the usual moral reflexes. As a reverse Rorschach, *White Lotus* anticipates the projection of conflict generated by a re-emerging class consciousness; as an entertainment, it plays on the ridiculous behaviours that result.

*White Lotus* can be superficially enjoyed as a critique of wealthy western hypocrisy; but its psychodynamically resonant setting and characters make it ideal for the practice of psychoanalytic interpretation, either alone or in a group setting, such as a psychodynamic film night.

